# Analectic

**Published:** 1838

**Authors:** 


### MISCELLANEOUS INTELLIGENCE.

### ANALECTIC, ANALYTICAL, AND ORIGINAL.

### I.—ANALECTIC.

### AD SYLVAS NUNCIUS.

1. *Treatment of Paralysis, at Paris, with the Actual Cautery.—New mode of Blistering.*

To the Editor of the Lancet.

SIR;—I beg leave to send to you a few remarks relative to the treatment at present adopted at Paris for the cure of paralysis, sicatica, and a variety of neuralgic diseases. Although the present mode of treatment of those diseases cannot boast of originality, still, its great success at *l' Hopital St. Louis*, may be a sufficient recommendation in offering it to your notice. I am, Sir, your obedient servant,

T. H. BURGESS, M. D., Edin., M. R. C. S. L.

11, Caroline-street, Bedford-square, Sept. 4th, 1837.

M. Jobert, who has revived the treatment of palsy by the actual cautery (as employed in the days of Hippocrates and Galen), had been rewarded by very great success in the wards of the above hospital, during the two previous seasons. The *Paralysis partialis* and *Paralysis venenata* of Cullen appear to be the types of this disease, from which the most beneficial results have been obtained by the use of the actual cautery. That species of paralysis which is produced by the poison of lead, or *paralysis saturnina*, has yielded almost invariably to three or four applications of the cautery. I have seen several individuals at this hospital, who were perfectly cured by the application of that remedy, with whom a fair trial had previously been made of the old régime, without any apparent advantage.

The treatment of paralysis by the actual cautery, is merely a revival of an old remedy, like "the treatment of hydrocele by incision," of which I gave you an account in THE LANCET, for the 27th of May last.

We know that with the ancients, the cautery was considered as a sovereign remedy for a variety of diseases. It has been employed to stop bleeding vessels. It has been employed for the destruction of carcinomatous tumors and ulcers, fistulæ, and polypi. It was principally relied on in chronic affections, as dropsies, diseased joints, palsy, &c. The implicit faith which was placed in this remedy, in the days of Hippocrates, may be perceived from the following aphorism:—Quoscumque morbos, medicamenta non sanant, ferrum sanat; quos ferrum non sanat ignis sanat; vero ignis non sanat, insanabiles, existimare, opportet." Even at the present day its efficacy in stopping the progress of hospital gangrene is admitted by all, and that, too, when all other remedies have failed in producing any effect.

The first individual on whom I saw M. Jobert try this remedy, was a young man, aged 22, a painter. He came into the hospital without possessing the slightest power in his left arm, which, when raised from the side, in a horizontal direction, and let go, fell down immediately to its original situation, and the patient evidently had not the power of even moving it again from his side. The arm and forearm were deprived both of motion and sensation; the temperature of that arm was below that of the right arm. It looked "shadowy, shrunk and weak," and, from the puckering of the skin, seemed to be gradually wasting away. Such was the state of this patient when he came under M. Jobert, before which he had been treated with several other remedies, among which were the favorite arnica montana, and strychnot nux vomiica, but without any decided advantage. A few days after he was admitted to the hospital, the *ferrum candidum* was applied by M. J. in the following manner: He commenced by gently drawing the red hot iron from the superior boundary of the disease down along the inner side of the deltoid muscle, so far as its insertion, that is, about the middle of the arm, or a little above it. He then pursued the same course with regard to the external margin of the same muscle, and united these two lines by two or three transverse sections, drawn across the deltoid, from its origin to its insertion. By this method, the powerful stimulant was made to operate along the course of some of the principal branches of the brachial plexus which go to supply the upper extremity. It passed, in part, over the tract of the internal and external cutaneous or perforans casserii nerves, operating on the latter near the insertion of the deltoid, where it is surrounded by the origin of the brach. anticus, and also on the external branch of the median or brachial nerve, which rises in common with the external cutaneous, and endows three of the fingers with sensation.

After the application of the cautery, or, rather, during its application, some spasmodic twitchings were apparent in the hand and fingers, and it is a curious fact that these spasmodic movements appeared to affect the ring finger much more than any of the others. Perhaps this was owing to its being supplied by branches from two distinct nerves, the median and ulnar, whereas the others are supplied solely by the median, on their palmar aspect. At least this is the only physiological explanation that can be given, unless we borrow the theory of the ancients, who supposed that this finger in particular was supplied with a vein, a special messenger, from the heart, and thereby endowed with a *higher degree of feeling* than any of its neighbors, which rendered it sacred to the marriage ring;—from whence its name, "Digitus annularis*"

After the application of the cautery, the surface over which it had passed was dressed with linen, previously steeped in oil, being applied merely for the sake of sheathing the seared surface, and preventing it from rubbing against the clothes The cautery was not allowed to rest on any part over which it passed *so as to penetrate deeper than the surface,* the object in view being merely to produce a shock on the nerves of the extremity, which can be sufficiently effected by searing the integuments along their course.

The first application of the cautery to this patient was on a Tuesday, and on the following Saturday it was again applied, in the manner above described. During the interval, the extremity was more or less subject to those convulsive twitchings which, by the way, were very unlike the *creeping*, *jerking* and *teasing* sensations (being so described by the patient) which are produced by the use of the strychnine, the spasms produced by the application of the *ferrum candidum*, being of a much more convulsive nature, and not continued. The patient said that the idea of horror (to use his phraseology) which arose in his mind when about to have a burning iron applied to his skin, was much more terrible and painful than the actual application of the object of fear; and after the first day he always allowed the cautery to be applied without any of that shrinking and shuddering which so harrassed him before the first application. In this case, this remedy was had recourse to five several times, and the patient was discharged, cured, on the twenty-sixth day from that on which he was admitted to the hospital. I observed this case with peculiar interest, from the first day, until the cure was effected. It was astonishing how soon, after having recourse to the actual cautery, he began to stir the arm that was before immoveable; and on the day before leaving the hospital he raised his hand to his head with as much facility as he could elevate the unaffected one.

During the period in which the above-mentioned patient was being operated on, a young woman, æt. 24, um "*conturiere*," was admitted into the hospital, her passport being a severe attack of sciatica. Her case immediately attracted the attention of the bold, and skilful, and enterprising M. Jobert, who looked upon her as a fit subject for the omnipotent iron. Here was a woman, young, handsome, and, excepting this neuralgic complaint, in the bloom of health; and it would appear, perhaps, revolting and cruel in this case to have recourse to a remedy which would seem to be even worse than the disease itself. She was asked, she was entreated, by M. Jobert, to submit to the operation; but no; she revolted with horror at the mere idea; and was backed by the *alma soror* who was matron of the ward. She remained in the hospital for ten days before she would even listen to its distinguished surgeon on this topic. At length, however, after much per-

*Of course, this theory existed long before the days of Harvey, and his discovery of the circulation of the blood.

suasion, she consented to allow him (as she said) to *touch* her with the iron. Being placed, face downwards, in her bed, M. Jobert proceeded to apply the cautery, having taken it red hot from a pan of burning charcoal, which is usually carried by a *garcon* for that purpose, when her whole frame quivered from head to foot, although untouched by the instrument. So great was the motion of the body, that the surgeon could not apply the iron for some time. After her agitation was somewhat abated, the cautery was applied lightly over the sacral and cocygeal regions; from thence, along the glutœus maximus, directly over the gemini and quadratus muscles, in a line parallel to the course of the sciatic nerve. During the entire period of the operation the patient never screamed, or seemed to feel much pain. She said, after it was over, that, had she known how slight would be the pain that it would produce, she would never have hesitated in permitting the cautery to be employed. It is merely the idea of having a red hot iron applied to the skin that deters and makes people shrink from the proposal; whereas, the actual pain that it produces, is not more than what is caused by a common burn. This young woman was cured after the third application, and discharged.

The iron should not, however, be used in that species of paralysis which is produced by "ramollissement," or softening of the medulla spinalis; but it might be used with advantage in that kind of palsy which is combined with rheumatism, and of very frequent occurrence in the lower ranks of life in our country.

In the French provincial hospitals, particularly in Normandy and Britany, paralysis of the extremities is treated by *vesication*, which is effected by a rather curious and novel method. The vesication is produced on the extremity affected, in the same relation with regard to the nerves, as the stimulus produced by the actual cautery above described.

The blister is raised in the following manner:—The surgeon cuts a piece of brown paper of the size and shape of the surface which he intends vesicating. This being well damped, or moistened with water, is placed on the limb affected, and a *smoothing-iron* (such as is used by washer-women), being previously *well heated*, is applied over the moistened paper; this plan produces a vesicated surface almost instantaneously, being effected by the steam generated by the contact of the hot iron and moistened paper. This method of blistering, being more speedy and *less painful* than that commonly adopted, is now generally used there in all cases where it is a matter of importance to produce immediate vesication.

---

2. *On Sedative Poison.* By Prof. Thompson.—Sedative poisons are usually ranked with narcotic poisons; but the difference of the effects produced by their influence upon the system, is too obvious not to point out the necessity of separating them, and forming a distinct class, or *sedative* poisons.

Sedatives, when administered in doses sufficient to prove poisonous, cause no previous symptoms of excitement: their depressing power is immediately felt, and often, in a few seconds, death, without any morbid symptoms, is the result. That this is the consequence of a direct sedative influence on the nervous energy is undoubted; and the fact that convulsions sometimes display themselves, when the dose is not sufficient to cause instantaneous death, is no argument against the opinion, since defective stimulus of the brain produces convulsions as decidedly as over-excitement of that organ; a fact strikingly illustrated in profuse hæmorrhages and over blood-letting.

The influence of sedative poisons is exerted chiefly on the nerves of sensation; and this is often displayed locally. Robiquet continued for some time to hold his finger on the mouth of a bottle containing some strong hydrocyanic acid, whilst he was conversing with a friend; it became numbed, and continued so for thirty hours, yet he felt no other inconvenience from the circumstance.

The sedative poisons differ in other respects from the narcotic; thus post-mortem examinations display no characters of inflammatory action having existed in the mucous membrane, or in the brain, or in the spinal chord; indeed no changes appear in either the stomach or the lungs, nor in any of the nervous organs.

The cause of death, in every instance, seems to be an immediate and direct exhaustion of nervous excitability.

### HYDROCYANIC ACID.

The first of the poisons is *hydrocyanic acid*, either alone or in substances in which it abounds; namely—the oil of bitter almonds, the distilled water of bitter almonds, the distilled water of the prunus laurocerasus, and noyau.

Hydrocyanic acid has too frequently been employed as an instrument of death by the suicide; occasionally, also, it has been used by the murderer.

In treating of poisoning by this acid, I do not intend to make any reference to the anhydrous acid; for, although it is the most virulent of poisons, yet, owing to the difficulty of keeping it, the possibility of its becoming a poisonous agent is very rare: it is the diluted acid, such as is usually employed as a medicinal agent, which demands our attention.

Many instances of its employment as an instrument of suicide are on record. Hufeland relates the case of a thief who swallowed f℥j. of the medicinal acid, equal to 40 minims of the anhydrous: he staggered, and died, in less than five minutes. A medical man, with whom I was intimately acquainted, determined to destroy himself. After making his will, and arranging all his affairs, he purchased an ounce of hydrocyanic acid, at Garden's, the Chemist, in Oxford-street; and walked as far as the end of Argyle street. Having called a hackney-coach from the stand, whilst it was turning round, he opened the phial, and drank its contents. He almost immediately staggered, was forced to support himself by holding by the post, and had scarcely power, with the assistance of the cad, to enter the coach, and order it to go to Sloane-st.; on arriving there, the unfortunate gentleman was found, seated in the corner of the coach, completely dead.

An instructive case of poisoning by this acid, where the person recovered, is detailed in the "Revue Medicale," for 1825. A physician at Rennes, who had taken two tea-spoonfuls of the officinal hydrocyanic acid with impunity, swallowed, on the 5th September, 1824, at seven o'clock in the evening, an equal dose of the acid twice at a very short interval. He had taken a hearty dinner five hours before. He had scarcely left the room where he swallowed the acid, when he felt a confusion of head; but as he had occasionally experienced the same thing before, he did not particularly attend to it. On entering his laboratory, he fell down like a person who had been struck with lightning, or with apoplexy. He lost all consciousness; his face and neck swelled; the pupils became fixed and dilated; trismus supervened; he scarcely breathed; the extremities were icy cold; a strong odour of the acid issued from his mouth; and the pulse was nearly imperceptible. In a few minutes, violent convulsions displayed themselves, and the extremities were horribly distorted. These symptoms continued for nearly two hours and a half, at the end of which time he gradually regained his consciousness; but several days elapsed before he completely recovered.

The French Codex, orders a syrup of hydrocyanic acid. It was administered in drachm doses to seven epileptics in the *Bicetre*, and all of them died, with symptoms similar to those which I have just described.

In whatever manner it is prescribed or administered, either therapeutically or as a poison, hydrocyanic acid operates directly on the nervous centres.

Experiments on the lower animals demonstrate that it acts most energetically when it is applied to serous membranes,—next, when it is taken into the stomach,—thirdly, on the cellular tissue and ulcerated surfaces,—and lastly, on the sound skin.

Emmert, Coullon, Krimer, and others, have drawn opposite conclusions to mine, with regard to its action on the nerves,—in consequence of observing that it does not act on cut nerves, nor on exposed nerves;—nor on the brain, nor the spinal chord. They also contend that, because when vessels of a part are tied, before touching it with this poison, its action is prevented; and it is not prevented by dividing nerves;—and because it is discovered in blood, it must necessarily operate by absorption.

The rapidity of its action,—for instance, the application of the strong acid to the eye of a dog, causing instant death, is against this idea:—and, in my opinion, it can only be explained, by supposing that the poison acts through the nerves.

The effect of a strong dose of hydrocyanic acid is death, almost instantaneous, and without convulsions. Such large doses may cause death in a few seconds; but some minutes may elapse.

In more moderate, but yet poisonous doses, the symptoms are vertigo, nausea, great depression of the strength; the weakness is felt chiefly in the limbs;—tetanic convulsions; gradually increasing insensibility, coma and death. In some instances salivation has supervened.

When it proves fatal, death generally takes place in less than fifteen minutes:—when recovery ensues, the symptoms may go on for many hours;—in some instances they have extended to twelve hours.

If a patient survive thirty or forty minutes, he will generally recover.

Nevertheless the symptoms have continued for more than a day, and yet death has ensued. In man, the period depends on the extent of the dose; but in no case are the fatal effects so rapid as in the lower animals. I have seen a dog, to whom ʒiv. of the medicinal acid were administered, die before he could be placed on the ground, from the lap of the assistant who held him. Some experiments were made by Mr. Macauley, of Leicester, to determine whether an Apothecary's servant, who was poisoned with hydrocyanic acid, could, after taking the dose, before becoming insensible, have time to cork the phial, wrap it up, and adjust the bed clothes. He made three experiments on dogs: one dog, who took fʒiv., died in four seconds; another, who took the same quantity, died in seven seconds; and a third, who took fʒivss., died in three seconds. As far as this evidence went, the presumption was, that the girl had either been poisoned, or that some one had been present to arrange these matters; for had convulsions taken place, her position would have displayed it. The apprentice of her master, by whom she was pregnant, was tried for the murder, but he was acquitted.

Such is the influence of this poison on the animal system.

CYANIDE OF POTASSIUM,

Which is formed by decomposing ferrocyanate of potasse, by a long continued red heat, separating the soluble matter, and evaporating to dryness, operates as a poison in the same manner as hydrocyanic acid. In a solid state this cyanide is deliquescent, very soluble, and has an alkaline reaction. It is inodorous, but has a taste resembling that of hydrocyanic acid. Its effects on the animal economy are exactly the same as those of hydrocyanic acid. It has been recommended to be used in the same manner as the hydrocyanic acid; and requires the same precautions.

OIL OF BITTER ALMONDS.

This is procured by the distillation with water of the marc remaining after the pressure of the bitter almonds, in order to extract the bland oil. It contains two substances which operate differently. One is an azotised substance, very poisonous; uncrystallisable; the other, unazotised, and readily crystallising on exposure to air or oxygen gas, which changes it to an acid.

The azotised substance exerts a powerful action on the animal economy, one drop almost instantaneously destroying a small bird.

On quadrupeds it exerts a less powerful action than hydrocyanic acid. Robiquet and Villerme tried its influence on a guinea-pig; one drop appeared at first to produce no effect, but after two minutes the limbs of the animal became tottering, the head swayed from right to left, the animal squatted on its rump, and described an arc of a circle, of which the dorsal line was the verge. To this succeeded convulsions of the thoraric muscles, and, after four minutes, it became calm. At the end of seven minutes the convulsions recommenced, the respiration was greatly affected, with a powerful contraction of the expiratory muscles. In three minutes more, every motion had ceased, and respiration was imperceptible, at the end of thirteen minutes the pulsations of the heart were more manifest and easily accelerated, but at the end of eighteen minutes every evidence of life was extinct. When the convulsions ceased, the muscles were relaxed.

The following are the usual symptoms of poisoning by oil of bitter almonds. In small doses, nausea and vomiting first occur, then diarrhœa and vertigo. In large doses, vertigo almost instantly comes on; the features are spasmodically contracted, the eyes are fixed and turned upwards; the pupils are immoveable; the breathing is stertorous; the pulse reduced to thirty in a minute; the breath exhaling the odour of bitter almonds; and death generally takes place in ten minutes.

A woman gave a child the expressed juice of a handful of bitter almonds to cure worms: colic, swelling of belly, vertigo, locked jaw, frothing at mouth, convulsions, insensibility, and death, happened in two hours. The poisonous influence of bitter almonds was known at a period so early, that before the time of Dioscorides, who mentions it, these almonds were used by the Greeks to kill wolves. According to some experiments of Orfila, twenty bitter almonds will kill a dog in six hours, if he be prevented from vomiting by a ligature on the œsophagus; and when six of the almonds were made into a paste, and introduced into a wound, they killed a dog in four days.

The volatile oil, procured by distilling the marc of bitter almonds, was experimented with by Sir Benjamin Brodie, who found that a single drop of it, placed on the tongue of a cat, killed it in five minutes. The bitter almond, when eaten in great quantity, causes nausea, vomiting, diarrhœa, and other symptoms of disordered digestive organs: and similar effects are produced, in some persons, even when a single bitter almond is eaten. In some cases an eruption resembling urticaria is produced. This was the case with the distinguished author of the "Conspectus Medicinæ," who was so susceptible in the digestive organs, that the same effect was produced on him by the white of an egg. A stout laborer had eaten a large quantity of bitter almonds. He dropped down, and Mr. Kennedy, who was sent for to see him, found him insensible; the pulse was imperceptible, and the breath exhaled the odour of bitter almonds, the remains of many of which were found in the stomach.

The effect of the oil of bitter almonds on the human subject is modified by the volatile oil to which it owes its odour, and which operates as an excitant, so that the influence of this oil more closely resembles that of a narcotic than a simple sedative. A gentleman, forty-eight years of age, swallowed ℨij. of it: his servant found him with his features spasmodically contracted, and his breathing hurried and convulsed. A physician who saw him twenty minutes afterwards, found him insensible, the pupils immoveable, the breathing stertorous, the pulse beating only 30 in a minute, and the breath smelling strong of the oil. He died ten minutes afterwards.

PRUNUS LAURO-CERSASUS.

Many parts of plants, which have the odour of the bitter almond, namely, the peach leaves, cherry-stone kernels, and the leaves and kernels of other species of prunus, are as poisonous as hydrocyanic acid, to which all of them owe their poisonous properties. The most poisonous of them, however, is the prunus *lauro-cerasus*, or cherry-laurel. It is a beautiful evergreen, flowering in July.

It is a native of the shores of the Black Sea, and was transported into Europe in the 16th century. It thrives freely in our gardens. The plant rises from five to fifteen feet in height, is very branching, and the stem is covered with a blackish bark. The leaves are almost sessile, distick upon the branches, oblong, acuminated at the apex, slightly denticulate, green, and shining on the upper disk, paler below, and ceriaceous. The flowers are in long, axillary racemes, each flower being supported on a short pedicel: they are white, small, and exhale a powerful odor. The fruit is an ovoid, elongated drupe, resembling the small black cherry, or guigores, but they are smaller. Their taste is sweet and insipid.

When the leaves of the plant are bruised and distilled, they yield an oil closely resembling that of bitter almonds, and the water which comes over with it contains a small portion of the oil, and a large quantity of hydrocyanic acid.

Dr. Price, the alchemist, destroyed himself with this laurel water, as it is termed. It was then frequently used as a sedative. Many experiments, made on quadrupeds, have determined its powers. Among other experimentalists, M. Ollivier, of Angers, found that f℥iv. of it will kill a strong dog in ten or fifteen minutes; and what is *a priori*, curious, the same dose produces the same fatal effect after it has been freed from the hydrocyanic acid, by means of potassa and sulphate of iron. This has been explained by the fact that, besides the free acid, it contains the elements of the acid, for when liquor potassæ is added to the fluid freed from the acid, and the mixture heated, cyanide of potassium is formed, capable of again forming Prussian blue with sulphate of iron.

As the leaves of this plant are often used to flavor custards and puddings, accidents have occasionally followed its use in this way. Dr. Paris mentions an instance of several children, at an English boarding-school, having suffered from a custard flavored with these leaves. Foderé notices, in his "Medicine Legal.," tom. iv., p. 27, the following fatal influence of it on two maid-servents at Turin, in 1784. These girls stole a bottle of the distilled water of cherry laurel, and, fearful of being discovered, they finished the whole in a short time, and almost immediately perished in convulsions. The bodies were opened in the University: the stomachs were found slightly inflamed, but the rest of the organs were in a healthy condition.

### SEDATIVES.—CHEMICAL TESTS AND POST-MORTEM APPEARANCES.

In every instance of poisoning by any of these sedative plants, or by the oil of the bitter almond, or by hydrocyanic acid, the post-mortem appearances are the same; and the mode of testing is in all cases the same as if the poison were simple hydrocyanic acid.

With regard to the post-mortem examination of the body in cases of poisoning by hydrocyanic acid, or the substances containing it, much depends on the period at which the inspection is made. The sooner it can be done with propriety the better; for when the body has been exposed for two or three days to the air, no traces of the poison can be detected in it. Its decomposition in the animal body is much hastened by the commencement of putrefaction, consequently the inspection should be made sooner in summer than in winter.

When death has taken place from hydrocyanic acid, the eyes remain glistening, and the features are as composed as those of a living person, appearances which continue for eight or ten hours; and for at least an hour after death, the eye is susceptible of the impression of light: the pupil dilates and contracts. I observed this very remarkably displayed in the case of the medical gentlemen which I detailed to you: the glistening continued for upwards of ten hours. It must be remarked, however, that this condition of the eye cannot alone be relied upon as characteristic of this description of poisoning, for the same effect is perceived in asphyxia from carbonic acid gas. Dr. Christison remarks, that he observed it six hours after death, in a woman who died of cholera. There is, also, a peculiar brilliancy in the eye in fatal instances of poisoning by strychnia, and after death from the epileptic paroxysm. This state of the eye, however, gives value to the other appearances.

With respect to the external condition of the body, the spine is generally stiff, and the belly drawn in.

On opening the body, the odour of the acid is frequently, but not always, perceived; when it is obvious it pervades every cavity; and, often, it is very perceptible in the blood, especially when the dose has been large.

On opening the *stomach*, the odour is often very obvious there, when it is not so obvious in the blood. This was the case in an instance recorded by Mertzdorff, and also, in the seven epileptics poisoned by the syrup of hydrocyanic acid in the *Bicetre*. In the case of the thief recorded by Hufeland, the villous coat of the stomach was red; and easily detached, as if in a gangrenous condition. I have never seen such an appearance in this viscus. It is a curious fact, that even when the odour of the acid has been very perceptible in the stomach, it has not been detected by the nicest tests; at least such was the case in instances opened by Coullon and others.

In the *intestinal canal* I have not been able to detect any change, although, in Hufeland's case, the mucous membrane was faintly reddened.

The veins of the liver, the spleen, and the kidneys, are generally gorged with black, fluid blood, which sometimes exhales a strong odour of the acid. The fluid state of the blood is not, however, constant; in the experiments of Coullon, Itner, and Emmert, on quadrupeds, the blood was coagulated. In a case of poisoning with the oil of bitter almonds, recorded by Mertsdorff, the colour of the bile was altered to blue; but this is a solitary instance of such an appearance.

On opening the *thorax*, the lungs are found gorged with dark-colored viscid blood, to a degree sufficient to make the more depending parts resemble liver. In some instances, when the dose has been very large, the right cavities of the heart have been gorged with blood, and the left nearly empty, as if the circulation had been suddenly arrested.

In the *cranium*, the vessels of the brain and its membranes are also generally gorged, and the odour of the acid has been detected in the ventricles.

Throughout the whole body there is an unusual turgescence of the venous system, with a corresponding emptiness of the arterial.

Now, let us suppose a case of suspected poisoning by hydrocyanic acid, or any of the substances containing it, which have been brought before you, and that your assistance is demanded to ascertain the fact. The first circumstance to which your attention is to be directed is the history of the case, the symptoms, and the post-mortem examination of the body; the second act of inquiry is to determine the fact of the hydrocyanic acid being, really, the poisoning agent.

If any of the poison remain, it may be readily recognised by its odour and taste; the acridity is felt especially at the back part of the throat, and this is caused as much by the odour of the acid as by swallowing it. The suspected fluid being thus rendered obvious by smelling and tasting, is then to be tested with several re-agents.

1. Add to the suspected fluid a few drops of solution of potassa, the fluid will remain colorless; then drop in a solution of mixed proto-sulphate, and per-sulphate of iron: it forms, at first, a brownish precipitate, which gradually acquires a greenish-blue tint, and, on the addition of a little acid, a deep Prussian blue is precipitated. The only nicety in this process is to be certain that the solution of the sulphates of iron contain a proto-sulphate, as the per-sulphate alone produces no precipitate of Prussian blue; and, on adding sulphuric acid, the brown precipitate is re-dissolved, but no blue colour or precipitate is formed as when the proto-sulphate is employed.

2. Add to the supposed hydrocyanic acid a few drops of liquor potassæ, and then some solution of sulphate of copper; a yellowish-white precipitate will be thrown down, and will become white on the addition of hydrochloric acid. This is caused by the hydrochloric acid dissolving some oxide of copper, which is thrown down by the potassa. The white precipitate is cyanide of copper, which is formed as a white powder, very abundantly even when the hydrocyanic acid is in small quantity. According to M. Lassaigne, it demonstrates the presence of the acid in 20.000 parts of water.

3. To the supposed hydrocyanic acid add a solution of nitrate of silver; a white precipitate is formed, which is not soluble in nitric acid at 60 deg., but it is soluble in that acid at its boiling temperature. This distinguishes this precipitate, the cyanide of silver, from the chloride of silver. If the precipitate be abundant, when dried and exposed to heat in a tube, it will evolve cyanogen, which burns with a violet-colored flame. One hundred parts of the dried cyanide correspond to rather more than twenty-one of the acid, so that the quantity of the acid may be known. This test may also be thus employed:—Put a drop or two drops of the suspected fluid, very slightly acidulated with distilled vinegar, into a watch-glass, and immediately cover it with a plate of glass, the under surface of which, to the breadth of a pea, is moistened with a solution of nitrate of silver, formed by dissolving one gr. of the nitrate in 2.000 grs. of distilled water. If the drop of the solution of the nitrate remain transparent, no prussic acid is present; if it become clouded, or milky, that acid is present, provided, on placing it over a glass of ammonia, it become again clear, and that it retain, unchanged, its white color, when exposed to light.

When hydrocyanic acid is mixed with wine, beer, coffee, milk, or other substances, it causes no change in their appearance, unless the mixtures have been kept for some time, when it gives a deep-brown tinge to them, owing to the decomposition of the acid.

In searching for hydrocyanic acid in mixed or compound fluids, if the quantity remaining in the glass or the cup be a few drops only, it may be tested with the watch-glass and nitrate of silver; but if it be in greater quantity, or if we are examining the contents of the stomach, the best method is to distil with a gentle heat, in a large retort placed in a water-bath, and to which a receiver is adapted by the interposition of a very long tube. The materials in the retort must not be allowed to boil, and the receiver must be kept cool with ice. The product is then to be collected, and tested in the usual manner for hydrocyanic acid.

This method of proceeding is applicable to the substance of the stomach itself, the blood, and the secretions; but, in examining these, the process must not be delayed longer than thirty or forty hours after the death of the poisoned person.

It is often of great importance to ascertain the quantity of hydrocyanic acid present in the fluid which has operated as a poison, and nothing is more easy, provided the matters to be examined contain no chlorides. It is effected by simply precipitating the fluid with nitrate of silver, collecting carefully the precipitate, washing and drying it, and then weighing it accurately; every 100 grains of the dried cyanide of silver contain 20.33 of pure hydrocyanic acid.

If we have the oil of bitter almonds mixed with the contents of the stomach, or other matters to examine, then the strong odour of peach blossom at once leads us to the determination of the poison; the same process of distillation at a low temperature, and the same mode of testing the result, is required, as when we are operating upon the diluted acid. The quantity of the acid in the distilled water of bitter almonds, and in cherry laurel-water, may be ascertained by simply dropping in the nitrate of silver, as in the simple diluted acid.

Now, let us take a case of actual poisoning by a substance containing hydrocyanic acid, for instance *cherry laurel-water.* This was the poison used for the murder of Sir Theodosius Boughton, for which Captain Donnellan was tried and convicted. The evidence on the trial, as far as concerned the condition of the body, and the draught supposed to have contained the poison was painfully conflicting. It was proved, both by Lady Boughton and by others, that the draught had a powerful odour of bitter almonds. She remarked, that when Sir Theodosius, her son, was taking the draught, "he observed that it smelt and tasted very nauseous, upon which I said, 'I think it smells very strongly like bitter almonds." Dr. Rattray, who was examined as to the appearance presented by the body after death, was desired, in Court, to smell the bottle from which the draught had been poured. His answer was, "I know the liquid well; it is a distillation of laurel leaves, commonly called laurel-water." Dr. Rattray, also, in reply to a question respecting the smell of the contents of the stomach, said, "One could not expect any smell, but partaking of that general putrefaction of the body; but I had a particular taste in my mouth at that time, a kind of biting acrimony upon my tongue. And I have in all the experiments I have made with laurel-water, always had the same taste, from breathing over the water, a biting upon my tongue, and sometimes a bitter taste upon the upper part of the fauces." Dr. Rattray very properly, however, did not impute this taste to any thing but the ammoniacal salts evolved from the decomposing body. I have mentioned it to show you, gentlemen, the necessity of not permitting your imaginations to run away with your judgment on such occasions. Dr. Rattray had been making experiments with laurel-water, under the idea that the poison on this occasion was laurel-water, and, evidently, imagined the taste; for, as the body was not opened for two days after death, and was in a state of decomposition, it was not likely that the hydrocyanic acid, contained in the quantity of laurel-water administered to Sir Theodosius, would then have remained undecomposed in the stomach. In opening bodies soon after death, however, this is an important point to attend to.

Nearly the same opinions respecting the contents of the bottle being laurel-water, were also given by Mr. Wilmer, Dr. Ashe, Dr. Parsons, Professor of Anatomy at Oxford; and they were all of opinion, that from the fact of the smell of the laurel-water in the bottle, and from the symptoms, Sir Theodosius had been poisoned by laurel-water. They based their opinion upon the effects perceived in quadrupeds poisoned by laurel-water. The answer of Dr. Parsons is striking, in reference to his knowledge of the poison being laurel-water. He was asked, "You collect that from the similarity of the smell?" he replied, "We have nothing else to judge from but the similarity of the smell." Nothing can more clearly elucidate the great advantages which Medical Jurisprudence owes to Chemistry than this reply.

John Hunter gave his evidence in a manner calculated to throw some doubt on the fact of the poisoning; and although I think that the evidence generally bore out the conviction, yet I cannot concur with Dr. Christison's remark, that Mr. Hunter's evidence "does him very little credit." I will read it to you, prefacing the perusal by remarking that the symptoms were, in every respect, those that have usually occurred in cases of poisoning by laurel-water.

"I don't wish to go into that; I put my question in a general way.—The whole appearances upon the dissection explain nothing but putrefaction.

"You have been long in the habit of dissecting human subjects; I presume you have dissected more than any other man in Europe?—I have dissected some thousands during these 33 years.

"Are those appearances you have heard described such, in your judgment, as are the result of putrefaction in dead subjects?—Entirely.

"Are the symptoms that appeared after the medicine was given, such as necessarily conclude that the person had taken poison?—Certainly not.

"Is any certain analogy to be drawn from the effects of any species of poison upon an animal of the brute creation, to that it may have upon a human subject?—As far as my experience goes, which is not a very confined one, because I have poisoned some thousands of animals, they are very nearly the same; opium, for instance, will poison a dog similar to a man; arsenic will have very near the same effect upon a dog as it would have, I take it for granted, upon a man; I know something of the effects of them, and I believe their operations will be nearly similar.

"Are there not many things which kill animals almost instantaneously, that will have no detrimental or noxious effect upon a human subject; spirits, for instance, occur to me?—I apprehend a great deal depends upon the mode of experiment; no man is fit to make one but those who have made many, and paid considerable attention to all the circumstances that relate to experiments; it is a common experiment, which I believe seldom fails, and it is in the mouth of every body, that a little brandy will kill a cat; I have made the experiment, and have killed several cats; but it is a false experiment; in all those cases where it kills the cat, it kills the cat by getting into her lungs, not into her stomach, because, if you convey the same quantity of brandy, or three times as much, into the stomach, in such a way as the lungs shall not be affected, the cat will not die. Now, in those experiments that are made by forcing an animal to drink, there are two operations going on; one is a refusing the liquor by the animal, its kicking and working with its throat, to refuse it; the other is, a forcing the liquor upon the animal, and there are few operations of that kind, but some of the liquor gets into the lungs. I have known it from experience.

"If you had been called upon to dissect a body, suspected to have died of poison, should you or not have thought it necessary to have pursued your search through the guts?—Certainly.

"Do you not apprehend that you would have been more likely to receive information from thence than any other part of the frame?—That is the track of the poison, and I should certainly have followed that track through.

"Then, in your judgment upon the appearances the gentlemen have described, no inference can be drawn from thence that Sir Theodosius Boughton died of poison?—Certainly not. It does not give the least suspicion.

"Mr. Howorth.—Upon the symptoms immediately produced, after the swallowing of that draught, I ask whether, in your judgment and opinion, that draught did not occasion his death?—I can only say, that it is a circumstance in favor of such an opinion.

"That the draught was the occasion of his death?—No; because the symptoms afterwards are those of a man dying who was before in perfect health; a man dying of an epilepsy or apoplexy, the symptoms would give one those general ideas.

"Then you decline giving any opinion upon the subject?—I don't form any opinion to myself; I cannot form an opinion, because I can conceive, if he had taken a draught of poison, it arose from that; I can conceive it might arise from other causes.

"If you are at all acquainted with the effects and operations of distilled laurel-water, whether the having swallowed a draught of that would not have produced the symptoms described?—I should suppose it would; I can only say this of the experiments I have made of laurel-water upon animals, it has not been near so quick; I have injected laurel-water directly into the blood of dogs, and they have not died; I have thrown laurel-water, with precaution, into the stomach, and it never produced so quick an effect with me as described by those gentlemen.

"But you admit that laurel-water would have produced symptoms such as have been described?—I can conceive it might.

"Mr. Newnham.—Would not an apoplexy or an epilepsy, if it had seized Sir Theodosius Boughton at this time, though he had taken no physic at all, have produced similar symptoms too?—Certainly."

TREATMENT.—HYDROCYANIC ACID.

The treatment of poisoning by hydrocyanic acid and its compounds is not satisfactory, owing to the rapid action of the poison. When our assistance is demanded, and we arrive in time, ammonia and brandy are the chief remedies. Bleeding has been proposed; but Orfila, who tried it, found it of no use. A much more certain remedy is the affusion of cold water on the head and spine. Mr. Simeon, apothecary to the Hospital of St. Louis, proposed chlorine. It was tried before by M. Coullon, without any advantage.—*The Lancet.*

---

3. *On the Human Voice.* By Prof. Muller.—We never remember to have read a more happy application of the laws of mechanical science to the structure and physiology of living bodies, than that which Prof. Müller has effected in the account which he has given in the third part of his "*Physiologie des Menschen*" of the larynx and voice. He has detailed them there at too great length to admit of our translating the whole section of the work, though it would fully deserve it, could we spare room for it; but we shall endeavor, in as brief an abstract as possible, to give the most important results which he has arrived at, cleared of all the technicalities of music and mechanics, which can be spared, without rendering the description unintelligible.

The following are the proofs which he adduces, independently of those drawn from his experiments of the glottis being that part of the vocal tube by which the voice is produced:—1st. That when an opening is made in the trachea below the glottis, the voice ceases, and returns when that opening is closed—while an opening made above the glottis does not necessarily destroy the voice. 2nd. Magendie's experiments, showing that the voice continues when the epiglottis, superior vocal (or false) ligaments, and the top of the arytenoid cartilages have been removed. 3rd. The visible vibration of the inferior vocal ligaments, when the voice is heard, in animals whose glottis is exposed, and in men who have attempted suicide. 4th. The injury of the laryngeal nerves, which supply the small muscles acting on and modifying the aperture of the glottis. 5th. The possibility of producing sounds similar to those of the human voice, by blowing through the larynx when removed from the body, when the vocal ligaments have a certain degree of tension, and the glottis is open to a certain width, and this, when every other part, except the inferior vocal ligaments and their attachments, is cut away. Hence the glottis and its immediate limits, the inferior vocal ligaments, may be regarded as the essential vocal apparatus—the trachea and bronchi being analogous to the conducting pipe, or, as organ-builders call it, the boot of the pipe—and all the apparatus above the inferior vocal ligaments to the body or bell of wind instrument or organ pipes, by which the sound produced in the glottis, or by the vibrating tongue is modified. In birds there is a different arrangement of similar structures; the organ producing the voice is the inferior larynx, which is at the division of the trachea, and to which the bronchi serve as the conducting pipe, while the trachea, and the upper larynx which has no ligaments, perform the office of the pipe which modifies the sound produced at the lower larynx.

The vocal ligaments are rendered capable of regular vibrations, like those of membranes stretched at both ends, by being composed of the same peculiar elastic tissue as has been pointed out by Lauth, Schwann, and Eulenberg, as existing in the middle coats of arteries—the gamentum nuchæ—lig. flava of the vertebral laminæ—stylo-hyoid ligament—longitudinal elastic fibres of the trachea and bronchi, and many other parts of the body of man and animals. It is characterized by its yellowness, dryness, slight vascularity, and by its frequently giving off from the larger bundles, of which its fibres are composed, very fine branches, which, anastomosing with those from adjacent bundles, often form a kind of net-work. In chemical composition it is principally distinguished from the other fibrous, tendon-like or ligamentous tissues, by the difficulty with which gelatine can be obtained from it—and in physical characters, it is most marked by its extreme elasticity, which is permanent in it under almost all circumstances, even after it has been preserved for many years in spirit. But the vocal ligaments are not the only parts of the larynx in which it exists; the thyro-hyoid and middle crico-thyroid consists of it, and it is extensively distributed through the whole interior of the organ, arising principally from the inferior half of the angle of the thyroid cartilage, and thence radiating in fibres backwards, downwards, and a little upwards, forming a continuous membrane attached to the whole of the upper edge of the crycoid cartilage (except where the arytenoid cartilages play), and to the anterior angle and edge of the bases of the arytenoid cartilages. A somewhat increased thickness of this membrane at three parts forms the so-called middle crico-thyroid, and the inferior thyro-arytenoid ligaments; it forms the upper vocal ligaments, which are also connected to the lower by a thin layer of elastic tissue lining the venticulum Morgagni. The same tissue is found also in the lateral hyo-thyroid, thyro-epiglottic, and glosso-epiglottic ligaments; and if to these be added the longitudinal fibres of it in the membranous part of the trachea and bronchi, an idea may be formed of the great extent of walls fitted for vibrations and resonance in the neighborhood of the vocal organ.

The vocal ligaments are made to possess various degrees of tension, by the contraction of the several muscles acting on the cartilages to which they are affixed. Thus the crico-thyroid, drawing the thyroid nearer to the cricoid cartilage, increase their tension, while the arytenoid cartilages are fixed; and the posterior crico-arytenoid muscles effect the same change if the thyroid cartilage be fixed, and the arytenoid cartilages be coincidently approximated by the proper arytenoid muscles. According to the degree of tension produced by these muscles, the glottis becomes longer or shorter. It is made narrower by the approximation of the arytenoid cartilages by the proper arytenoid muscles, and wider by the separation of these cartilages by the posterior crico-arytenoid muscles.

The elasticity of the ligaments connecting these cartilages will maintain the corresponding edges of the thyroid and cricoid cartilages in approximation, without the aid of muscular effort; so that while the arytenoid cartilages are fixed, there will always be a certain degree of tension of the vocal ligaments; and when the posterior crico-arytenoid muscles act, to draw the arytenoid cartilages backwards, they must have this elastic middle crico-thyroid ligament to act against.

The glottis is capable of receiving the following forms:—When at rest it is lanceolate; growing wider on inspiration, and narrower on expiration. Its sides are formed behind by the inner surfaces and anterior processes of the bases of the arytenoid cartilages; before, and to the greatest extent by the vocal ligaments, attached to those processes, and the angle of the thyroid cartilage. When at its greatest width, it has the form of a lozenge, with its posterior angle cut off, and the distance between the lateral angles, at which are the anterior processes of the arytenoid cartilages, may be 5 3-4 lines. In its narrowed condition the glottis may have a triangular form; it may be open in its whole length, the arytenoid cartilages being merely approximated; or by their coming in contact at their anterior angles, a double aperture may be produced; or by their coming completely in contact, the posterior part of the glottis may be entirely closed, and there will then be no opening except that between the vocal ligaments. This last condition is produced by the united action of the lateral crico-arytenoid and the proper arytenoid muscles; the aperture will be pointed both before and behind, and will vary in width according to the coincident degree of tension of the vocal ligaments.

The loosening and shortening of the vocal ligaments is effected by the thyro-arytenoid muscles, which at the same time narrow the space above and below the lower vocal ligaments.

The exact form of the glottis during the emission of sound is not precisely known, but it is certainly very much narrowed, as is shown by Mayo's observations on those who had attempted suicide, Rudolphi's in a case of loss of the nose and palate, and Magendie's on living animals. Müller thinks, in accordance with the opinions of the last mentioned physiologist, that the posterior part of the glottis, between the arytenoid cartilages, is generally closed during the production of voice, but as in the dead larynx he does not find this a necessary condition, he does not so decide it.

The following is the mode employed by Müller in experimenting on the production of voice with the larynx after it is removed from the body. The principal objects to be attained are, a fixed position of certain of the cartilages, and at the same time a regular and measurable power of moving others, so as to give the vocal ligaments the desired degree of tension and varied forms. These he attains by laying the larynx with its posterior wall on a board, and there fixing it firmly by the cricoid cartilage; the arytenoid cartilages are then fixed by an awl or metal pin, run transversely through them both, near their bases. This is the most delicate part of the process, requiring great care, in order that when the extending force is afterwards applied, both vocal ligaments may be made equally tense; and so that when these cartilages are pressed towards each other on the pin, their anterior or vocal processes may touch. The transfixing pin may then be fixed with cords to the board. When this has been accomplished, and the cricoid and arytenoid cartilages are both fixed to the board, any degree of tension may be given to the vocal ligaments by drawing the angle of the thyroid cartilage forward. This is best done, and will admit of being measured, if a fine string be affixed to it just before the insertion of the ligament, then passed over a pulley, and then have hung on at its opposite end one of a pair of delicate scales, which may be loaded with different weights, so as to draw away the anterior part of the thyroid from the arytenoid and cricoid cartilages, as much as the vocal ligaments will allow. In proportion as the weight is increased, the vocal ligaments will now be stretched. All the parts of the larynx above the lower vocal ligaments may now be cut away, as they are unnecessary for the production of the required sound, and the actions of the ligaments may be better observed when they are removed. A wooden tube to blow through is put into the end of trachea.

The following facts have been observed in the frequent performance of experiments on larynges, thus prepared.

On blowing through the trachea when the glottis is narrow, the vocal ligaments give out full and clear notes, which come very near to those of the human voice, and much resemble those produced by blowing over moist elastic arterial coat, stretched over the end of a table, as in the best kind of artificial larynx. They differ from those obtained when the ventricle, upper ligament, &c. are affixed, only in being less loud. The vocal ligaments sound best and most easily when the posterior part of the glottis, between the arytenoid cartilages, is closed, though this form is not absolutely necessary, and if the tension of the ligaments be the same in both cases, the note produced is of the same height, whether the posterior part of the glottis be open or not, which is at once an evident proof that it is the vocal ligaments whose vibrations produce the sound, and that the air is not the primary vibrator, for in that case the note would be deeper when the glottis was open to its whole length, than when it was open only for the length of the vocal ligaments. And if the anterior angles of the arytenoid cartilages touch, so that there is an aperture both behind and before them, no second note is produced by the second posterior aperture, though the air sometimes rustles, in passing between the cartilages and their uniting membrane.

If the ligaments be equally tense in all cases, the width of the aperture of the glottis does not make any difference in the height of the note produced,—it is only less clear, in consequence of the noise of the air rushing through, when there is a wide opening; and in this, as in many other respects, a larynx exactly resembles the artificial ones made with bands of caoutchouc and instruments with membranous and metallic tongues, in which a wider opening only alters the expression, and not the height, of the note.

If the vocal ligaments be unequally tense, there are rarely two notes produced; but when the tension is equal, there is occasionally produced a much higher note than the proper one, from the ligaments coming in contact in some part of their length. Notes can as well be produced when the ligaments touch each other every where, as when they have a narrow opening between them; and if the tension be the same, the notes produced in these two conditions are not different as regards their height. In these cases the notes are produced most easily when the ligaments which are in contact are very lax, and they are caused by the interrupted passage of air through them, and may be well produced when the glottis is extremely short.

Low notes may be produced in a very short as well as in a very long glottis, and high notes in a very long as well as in a very short one: if only in the short one for low notes the ligaments are very lax, and touch each other; and if in the long glottis for high notes, the ligaments are at the same time very tense. This shortening and lengthening of the glottis, without alteration of its tension, may best be effected by compressing the lips of the glottis with forceps; while the degrees of tension may be altered by pressing the angle of the thyroid cartilage backwards to the cricoid.

When the whole vocal ligaments, from the angle of the thyroid cartilage to the vocal processes of the arytenoid cartilages (in contact with each other), are in vibration without touching, the notes produced by increasing degrees of tension of the ligaments do not increase in height in precisely the same degrees as those produced by strings and membranes stretched at both ends; there are always some half or whole notes below those given by strings and membranes. For example: with the same length of string, the notes or numbers of vibrations increase directly as the square roots of the powers producing the tension; i. e. if a string, extended by a weight of 4, give the note *c*, it will give the octave or *c*' when extended by a weight of 16, and the second octave or *c*'' with a weight of 64. But by adding weights into the scale attached to the thyroid cartilage, as above described, according to this rate of increase, octave's are not obtained from the vocal ligaments, but notes, 1-2 a note, or 1, 1 1-2, 2, or 3 whole notes below the octaves. However, the analogy is near enough to show that the notes of the human vocal organ, so far as they are produced by the glottis and its limits, are analagous to those of strings and membranous tongues. [The experiments proving this are of very great difficulty, and require considerable practice to perform them correctly, but we cannot undertake to translate the whole of Müller's long directions.]

*London Medical Gazette.*

---

4. *New Experiments of the Sense of Taste in Man.*—In 1830, MM. Guyot and Admirault published a series of experiments on the seat of taste in man, from which they drew the two following conclusions:—

1. The lips, the inner part of the cheeks, the roof of the mouth, pharynx, velum palati, dorsal and inferior surfaces of the tongue, have no share in the production of taste.

2. The sense of taste exists only at the posterior part of the tongue; along its edges for about a line or two towards the dorsal surface; at the point of the tongue; and, finally, at a restricted point of the velum palati, situate very nearly at the centre of its anterior surface.

In a second memoir, lately published by the same authors, an additional number of experiments has been recorded, together with a solution of the following questions:—

1. Do the gustatory surfaces perceive, with an equal degree of intensity, throughout their whole extent?—No. Taste is much stronger at the base of the tongue, and along its edges the gustatory power goes on increasing from the pillars of the velum pelati up to the tip of the tongue, where it is at a maximum.

2. Do the gustatory surfaces perceive indifferently all kinds of savors? Certain bodies, such as milk, butter, oil, and especially alimentary substances, only produce an impression of tact, at the anterior part, their characteristic tastes being only distinguished at the posterior part of the tongue.

3. Does a sapid body produce an identical taste when applied to different regions of the tongue?—A great number of bodies, and salt in particular, exhibit this very remarkable phenomenon, that the sensations which they produce at the anterior and posterior parts of the tongue are extremely different. Thus, solid acetate of potass, which, at the anterior parts of the tongue produces a burning acid sensation, is merely bitter and nauseous when applied to the posterior surface, upon which it produces no acid nor stimulating impression. Sulphate of magnesia, slightly acid and saltish in front, becomes intensely bitter at the root of the tongue. Acetate of lead produces only a sweet taste at the posterior part; anteriorly it is styptic, fresh and stimulant. The alkalies, the water of lime, and of ammonia, produce only one taste, no matter where they may be applied.

From the very numerous experiments which M. Guyot has performed, both on himself and on other individuals, he concludes—

1. That taste is a physical and not a chymical sense: that it is connected with bodies, and not with their densities, their temperature, or their consistence. In this respect it differs very considerably from the senses of touch or tact, which are exclusively destined to recognise the physical properties of bodies.

2. That the sense of taste must be exercised by at least two nerves.

Numerous anatomical investigations have led M. Guyot to regard the glosso-pharyngeal nerve as the one which presides over the perception of taste at the base of the tongue, and perhaps at the velum palati; while the lingual nerve, on the contrary, exercises the sense of taste at the point of the organ.

In this point of view the sense of taste, reacting on the glosso-pharyngeal nerve, probably determines the acts of deglutition and regurgitation. By the communications and terminations of this latter, it may act simultaneously on the amygdalæ, provoking their secretions; on the glosso-staphyl, which aids in closing the glottis and epiglottis, &c. &c. In the same manner the lingual nerve, receiving sapid impressions at the point of the tongue, may provoke contractions in that organ, and, in a word, harmonize and complete the function.

*Gazette Med. de Paris.*

—

5. *Severe Epileptic Convulsions cured by compressing the Carotid Artery.* By M. Trousseau.—On Monday, the 11th of September, 1837, M. Trousseau was called to see, in consultation with Messrs. Chomel, Cerise, &c., a child of eight years, laboring under violent convulsions. The patient had had scarlatina in August, and shortly after, having been exposed to cold, he became œdematous, but only slightly so. The œdema had diminished on the 10th. In the evening the child complained of the head, and he passed an uneasy night. On the 11th he was unusually loquacious; vomited several times, and had most severe pain in the head. At three o'clock a violent fit of convulsions set it; ten leeches were applied to the ears, and sinapisms to the legs. A second attack came on shortly after the first, and when M. Trousseau arrived, he found the child stretched on the bed, with the head firmly thrown backwards; the jaws, eyelids, muscles of the neck, the arm and leg of the right side of the body were agitated with violent convulsive movements; the left side of the body lay quite tranquil; the pulse was tumultuous, 160; the pupils excessively dilated, and the breathing become stertorous. Under these circumstances, M. Trousseau proposed the cold effusion, not to exceed half a minute in duration. This was administered, but without effect. The convulsion had now lasted two hours, and life was fast ebbing away, when M. Trousseau conceived the idea of impeding mechanically the flow of blood to the brain. He, therefore, applied pressure to the carotid artery, but, in the hurry of the moment, selected the right carotid. The convulsions continued, and two minutes elapsed before he was aware of his error. The index-finger was now immediately placed on the left carotid artery, and in about fifteen seconds the convulsive movements suddenly ceased, the child falling into a kind of apoplectic stupor. Compression was kept up for an hour more, and, at the end of that time, the state of the child was very satisfactory. The sensibility, however, was perfect on the left side of the body, but was very dull on the right side, which lay completely paralysed. The compression was kept up at short intervals, and a drop of croton oil given, to act as a derivative. The child was somewhat uneasy during the evening, and manifested signs of cerebral excitement, but the convulsions did not return; the paralysis of the right side also disappeared in the night, and on the following day the child was very well. His convalescence was rapid.—*Jour. des Con. Med. Chir., October*, 1837.

—

6. *Poisoning with Cream of Tartar.*—John Hudson, æt. 37, residing at 24, Southampton-street, Euston Square, employed by the notorious and self-styled *Dr.* Morison, at his pill establishment in the New Road, King's Cross.

On Tuesday evening, Oct. 10, he returned home extremely weak, and scarcely able to walk. He stated that he had been severely purged, had at least had twenty-five motions, attended with constant vomiting. On the previous day (Monday) he was drunk. The nurse told me, that one of Morison's men said he was sure Hudson had taken nearly a quarter of a pound of cream of tartar at one time, and that during the day he was continually putting small lumps into his mouth, to cool his stomach. The nurse also noticed a quantity of white substance at the bottom of the chamber utensil; she then asked him if he had been taking any salt. It was unfortunately thrown away.

11th.—At twelve o'clock he was seen by a medical gentleman, who found that during the night he had been repeatedly purged, and suffered greatly from constant vomiting. He complained of pain in the umbilical region, and of great thirst. Tongue brown and dry; pulse feeble; pain in the loins. The thighs and legs appeared paralysed. The fluid vomited was of a blackish green color, and the motions of the color of coffee-grounds. He stated that he had taken four or five table-spoonfuls of cream of tartar, which is a principal ingredient in Morison's cooling powders. An opiate was given to him, which afforded slight relief; but the symptoms returned, and he died on Thursday at noon.

14th.—I was requested to assist in making a post-mortem examination of the body. He was a strongly-built, well-formed man, stout and muscular; no spots or bruises upon the body. The fat covering the abdominal muscles about half an inch thick.

*Stomach.*—This was distended with gas, and contained about three ounces of a thick brown fluid, colored apparently with bile. Near the pylorus there were several red patches. The cardiac end of the stomach was very much inflamed, the mucous membrane being of a deep red color, with three or four spots of a blackish red, as if from rupture of some of the minute blood-vessels. The mucous membrane of the duodenum was also of a red color in many places, but not in so great a degree as that of the cardiac end of the stomach; it contained, apparently, the same kind of fluid as the stomach. A portion of the small intestines, as also the colon, had the mucous membrane reddened. The mucous membrane of the rectum was injected in small streaky red patches; where there was no redness, the membrane was of a white colour. The intestines contained a thick brownish kind of mucus; no fæcal matter was observable throughout their whole extent. A great deal of fat was attached to the large intestines, and the omentum contained a large quantity.

*Chest.*—The lungs might be termed healthy, though there were adhesions at the posterior part of the lobes of the left side, and at the anterior part of the lobes of the right, evidently of an old formation.

*Heart.*—A great quantity of fat about the pericardium. The heart was unnaturally large and flabby; the lining membrane of the left auricle was of a deep red color, as also the lining membrane of the aorta and semilunar valves. It weighed 13 oz.

*Liver.*—Large and very firm. On cutting it, an oily appearance was left on the knife, and oil could be scraped from the cut surface. It weighed 4 lb. 11 oz.

Spleen healthy. Kidneys large, and embedded in fat.

---

7. *Polypi in the Meatus Auditorus Externus.* By EDWARD J. DAVENPORT, M. D., BOSTON.

The lining membrane of the meatus, like the mucous surfaces of other cavities, is not unfrequently the seat of polypous excrescences. Professed works on the diseases of the ear afford, however, very meagre and unsatisfactory accounts of their origin, and the mode of treatment to be pursued for their removal. Polypi are referred, by the best authorities, to the occurrence of irritation or inflammation in the auditory passage or in the chamber of the tympanum, and are often found among the sequelæ of scarlet fever, measles &c. They consist chiefly of congeries of blood-vessels, loosely connected by cellular membrane, and possess a very low degree of vitality. Patients of a strumous habit, and those who have been exposed to a cold, moist atmosphere, are believed to be more particularly liable to these excrescences, as well as to other diseases of the ear. Recent polypi are of a soft and spongy consistence, of a reddish color, and bleed easily when injured. When first presented for examination, they are always found surrounded and partly concealed from view by a thin, purulent fluid, secreted by the diseased ceruminous glands, and probably by the surface of the excresence. The above-named glands take on a morbid condition from the constant irritation of the polypus, which acts as a foreign substance, and the increased and vitiated secretion is both a proof and effect of their presence. The discharge is not always thin, but varies in consistence, being sometimes thicker and more like pus, particularly when the disease is of long standing. It may, likewise, be fœtid and acrimonious.* After a time, the tumor will be found denuded of its cuticle or excoriated, and sometimes ulcerated. In those cases in which a portion of it protrudes beyond the limits of the meatus, and is exposed to the influence of the atmosphere, the extremity, being unprotected by the discharge, becomes somewhat hardened, and of a whitish color, similar to the cuticle of external parts.

Polypi of the ear have, in general, a narrow neck or peduncle, while the body of the tumor may nearly fill up the auditory passage, and diminish to a point externally, sometimes projecting 4 or 5 lines beyond the orifice of the meatus. In many cases, however, the origin of the tumor is equal in size or larger than the body, and then the latter assumes a conical form;† in others, there is no more than one tumor springing apparently from the same base. Polypous excrescences may grow from the parietes of the auditory passage, or from the surface or the edge of the membrane of the tympanum, but perhaps their most frequent origin is from the lining membrane of the chamber of the tym-

---

*Vide Buchanan's Illustrations of Acoustic Surgery.

†Sometimes they have the *form*, color, and consistence of a strawberry.—Vide Saissy on the Ear.

panum,* this cavity being frequently the seat of abscesses and purulent collections, after an attack of scarlet fever. In the latter case, the membrana tympani is uniformly more or less destroyed or perforated, and fluids injected into the ear will often pass into the throat, much to the annoyance of the patient.

As to the progress of polypi, under ordinary circumstances they are of slow growth, and they may exist for many years without impairing the general health. But not so with respect to the sense of hearing, which is always more or less impaired by the mechanical obstruction of the tumor, and most commonly is irretrievably lost by the ulceration of the surrounding parts and the destruction of the membrana tympani, and the consequent loss of the ossicula auditus. Cases, however, have been reported of the recovery of hearing, upon the removal of polypi of many years standing.† In these instances no doubt the tumors grew from the parietes of the meatus, and merely presented mechanical obstacles to the passage of vibrations of the air to the membrana tympani, without destroying the internal mechanism of the organ.

Among other causes of polypi, may be mentioned blows and falls upon the head, a spiculum of bone forming a nucleus, the frequent use of an ear-pick, causing irritation, &c.

In the treatment of this disease, as might have been expected, injections into the ear have been much resorted to. These have consisted of solutions of stimulating and escharotic substances, infusion of tobacco, &c. Their removal has also been attempted by repeated applications of caustic in a solid form, by the knife, by ligature, and, finally, by extraction or laceration. In children, and where the polypus is of small size and recent, a cure may usually be effected by the frequent application of a strong solution of sulphate of zinc—℈ij. to ʒi. to one ounce of distilled water; or it may be slightly touched with a camel's hair pencil dipped in the tincture of muriate of iron, once each day, until its removal is effected, after which the parts may be dressed with weak citrine ointment on soft lint. Extraction of the tumor has the merit of being at once effectual and perfectly safe. It is most easily accomplished with a pair of artery forceps. The patient being placed so that the rays of the sun will fall in a line with the direction of the meatus, and the external ear being slightly elevated and drawn backwards, so as to reduce the curvature of the passage, the forceps are to be firmly applied, as near as possible to the origin of the polypus; then giving the instrument a half turn and withdrawing it at the same time, the tumor is displaced and removed. The hemorrhage that follows the operation is not of serious consequence, and generally ceases spontaneously. In one case it is stated that nearly five ounces of blood were lost, and the patient was benefitted thereby. Usually the quantity is very much less than this. Should any portion of the excrescence remain, or a fungous growth take place, powdered alum may be applied daily; or a mixture of creosote with almond oil, in the proportion of one drop of the former to six of the latter, may be dropped into the meatus once or twice

*Vide Stevenson on the Ear.

†In one instance fourteen, and in another seven years.

each day. This latter remedy has the farther advantage of restraining any purulent discharge that may continue from the diseased state of the tube.

I subjoin an account of two cases in which polypi were removed by ligature. The first occurred in a young woman 18 years of age, of delicate health and pale complexion. Of her case I have the following notes. Six years previous to her application, she was attacked with slight otorrhœa in the right ear, for which she could assign no cause. During this period, the discharge had never ceased entirely, but a few months since, previous to the formation of an abscess in the meatus, it diminished greatly for a short time. It has varied from time to time in amount, quality, and consistence, being sometimes sanious and bloody, and at others more like true pus. The ear has not been generally painful, except during the formation of the abscess mentioned above. Hearing, in this ear, is nearly or quite extinct, but in the left ear it continues perfect, except when suffering from catarrh, to which she is subject. Upon examination there was found, filling up the meatus, a polypous excrescence of a pale red color, having its surface granulated somewhat like a mulberry, and its body divided into two portions, uniting at the base of the tumor in the chamber of the tympanum. The origin of the tumor was ascertained by means of a small, blunt probe. It possessed but little sensibility, but bled upon the slightest touch, even upon injecting the passage with simple water for the purpose of removing the copious secretion of purulent matter, with which it was enveloped. The lining membrane of the meatus was in a state of high inflammation, with increased sensibility and tenderness. The mother of this patient has been affected with otorrhœa, more or less, since childhood, and her hearing is impaired. The tumor was removed two years ago, with a flexible silver wire ligature introduced by a small eye probe, as near the base as possible. A slight hemorrhage followed the operation, but ceased spontaneously. Otorrhœa continued for a time, though much less than before. Hearing was not improved. At the end of five or six months, she returned with otorrhœa somewhat troublesome, and with occasional pain referred to the meatus. A fungus, of a granulated appearance and florid color, was now perceptible near the edge of the membrana tympani. Upon touching it with a pencil dipped in a saturated solution of nitrate of silver, it bled freely. Upon the use of creosote injections and the occasional application of a blister, all the unpleasant symptoms subsided.

Two months since, this patient had a slight return of otorrhœa from cold, which induced her to apply, from the apprehension of a recurrence of the polypus. Not the slightest trace, however, of this could be seen, and the otorrhœa was soon checked by the usual remedies.

The second case occurred in the person of C. M., a native of Germany, 28 years of age. In this patient there was a large, firm polypus in the left ear, with destruction of the membrana tympani and entire loss of hearing; and in the right ear, a troublesome otorrhœa with perforation of the tympanum, and the sense of hearing very much impaired. He had otorrhœa in the left ear since he was a child, and in consequence of cold and wet, while on his passage to this country two years ago, he experienced a severe attack of pain in head and ear, attended with increased discharge of purulent matter from the meatus. Soon after he perceived a polypous excrescence in the auditory tube, which remained about the same in size up to the time when he applied for advice. There has been a constant foetid discharge from his ear, occasional hemorrhage, more or less pain, and a buzzing *(bourdonnement)* or roaring noise, which equally affects both ears. The disease in the other ear he attributes to a violent blow on the right side of the head from a stick of wood, when about 16 years of age. This was followed by a discharge of blood mixed with pus, and at the same time a diminution of the power of hearing. The degree of hearing varies, however, at different times; thus when affected with catarrh, hearing is more imperfect; likewise when, from any cause, the discharge from the ear is materially diminished in amount, he does not hear so well as usual. This circumstance has been noticed in other cases where the membrane of the tympanum and the chain of little bones have been destroyed, and it is explained by the supposition that the matter acts in some measure as a substitute for these parts in conveying the vibrations of the atmosphere to the labyrinth, the immediate seat of the sense of hearing. In this ear the parietes of the meatus and the edges of the membrana tympani were much inflamed and thickened—not an uncommon state in chronic affections of the ear attended with purulent discharges. Injections passed from the ear into the throat, and air could be forced through the meatus. The polypus, which was somewhat firmer than usual, was removed by ligature. No bad conseqences followed the operation; but the hearing was not at all benefitted, when the patient changed his place of residence, and was not again seen.

In the case of a child, 6 or 8 months after an attack of scarlet fever, a polypus of the meatus was removed entire, which presented a narrow peduncle, with a broad root or base, by which it was attached within the chamber of the tympanum. In another case, of a young girl from the country, a polypus existed at the same time in each ear, the result of measles or scarlet fever. This patient retained a slight degree of hearing. Other cases have fallen under my observation, the particulars of which I cannot now recall to mind.

No. 4 Winter street, Nov. 1837.

---

8. *Intermittent Neuralgiæ.*—Dr. McPhail, of the army, has communicated to the American Medical Library and Intelligencer, five papers on the diseases that prevail in the south-western parts of the United States, under the title of Medical Topography, which show him to be a man of critical observation, a careful practitioner, and a desirable correspondent. Dr. McPhail's observations on the particular subject of intermittent neuralgiæ, constituting the fifth article of the series, is that which has most interested us, and this circumstance has led to the publication of the spirit of it in this place. He represents that *periodical affections, not strictly febrile,* are common in Arkansas. We should judge that they were painfully so from the remark that follows: "I have treated cases of intermittent frontal, facial, digital and articular neuralgia, cephalalgia, odontalgia, otalgia, gastralgia, hepatalgia and pneumonalgia. This last form of affection has, I believe, not yet been noticed by medical writers." Dr. McPhail describes this as so much resembling inflammation of the lungs, as to have deceived the physician, who, by adopting a treatment indicated by what was supposed to be the condition of those organs, well nigh brought on fatal results. He goes on to say, that neuralgia, depending upon the influence of malaria, may be easily distinguished from that having its origin in morbid alteration of the brain or nervous centres, the generation of tumors, &c. In cases of tic douloureux, depending upon malaria, the paroxysms are generally periodical, passing off speedily. Those, however, depending on organic changes, are characterized by an almost constant pain, remitting, or in fits of long duration. In those instances where the brain, nervous trunks or slender filaments, like the frontal or facial twigs of the fifth pair, or the digital branches of the median or ulnar nerves, have been the seat of the disease, the pain has ordinarily been quick, sharp and shooting. When the liver, spleen, or stomach were attacked, then the pain has been dull, gnawing, or having the sensation of burning. These are nice distinctions, not to be lost sight of by the practitioner, let his residence be where it may. Again, when the organs of reproduction are the seat, then the pains are lancinating. In the supra-orbital nerve, the true character of the affection may be recognized by a contraction of the pupil, in some individuals, and in some which came under this gentleman's eye, a sanguineous suffusion of the conjunctiva, with intolerance of light, and profuse lachrymation, were the accompaniments. If the infra-orbital nerve gives evidence of being affected, it is manifested by muscular twitchings; the same may be said of the sign of neuralgia in the facial threads of the inferior maxillary.

There is a necessity for passing over many paragraphs quite as interesting, evincing the writer's powers of investigation. The treament is an essential appendage to the foregoing epitome of Dr. McPhail's valuable communication. In those attacks of the small nerves of the head, face, and jaws, adverted to, if there is no appreciable disturbance of the general economy, in the interim between the attacks, to use his own expression, he gives "two grains of the sulphate, or a quarter of a grain of the arseniate of quinine, in pills, every two hours—with from six to twelve grains of the sulphate or one grain of the arseniate at the period of time next that of the expected paroxysm—always with the effect to prevent a recurrence." By pursuing this course till the terms of two or three paroxysms pass by, a pill of two grains of sulphate or phosphate of quinine or arseniate of quinine and piperine, or six or eight drops of Fowler's solution, twice or thrice a day, finishes the treatment.

In two cases of hysteralgia treated at Fort Jackson, below New-Orleans, on the Mississippi river, Dr. McPhail speaks of hysterical mania in its most frightf l form, complicated, in one instance, with dysmenorrhœa.

The scheme so fully and perfectly carried out in writing the medical topography of the region in which he is temporarily located, is very honorable to the author, and it moreover redounds to the character of the medical department of the army of the United States, that talent and learning, of so high an order, are to be found in the staff. The appeals which have been unremittingly made to the profession to study the character of diseases where they reside, the causes which produce them, the modes of treatment, and the result on the public health, seem to have been absolutely forgotton. However, we feel no less solicitous on this account, discouraging as it may seem, to persevere, and we shall continue, therefore, to urge upon the consideration of our readers, the claims of the people upon them to be active in the inquiry. How is it possible to obtain pathological facts of topographical value to a succeeding generation of physicians, if the present occupants of the soil contribute nothing towards the object?

*Boston Medical and Surgical Journal.*

---

9. *A case of Malformation of the Septum Narium.*—From youth up, persons with whom I conversed, often justly complained of my indistinct enunciation, and remarked that it was oftentimes impossible for them to understand many words I uttered. Being reminded of this defect by teachers and friends almost from the moment I was able to articulate the first words in childhood, until I had attained to maturer years, I made constant efforts to overcome the difficulty, and particularly in all words, which I had occasion to use, having the nasal sound, yet without any success. I was, therefore, greatly to my annoyance, compelled to sit as a silent listener to the conversation of others. It was not until the summer of 1830, that I began to suspect the real cause of all my trouble. From my then entire ignorance of the true *nature* of the difficulty, I was apprehensive of serious consequences, and consulted the most eminent neighboring surgeon. Not comprehending its character, his prescriptions tended to increase, rather than lessen, my affliction; for the local use of iodine ointment and tincture had not a very soothing effect on the delicate and sensitive surface of the schneiderian membrane. Great determination of blood ensued, and consequent thickening of its tissue. After this, the same surgeon advised me to have an incision made through the ala nasi to the nasal process of the superior maxillary bone, and then the offending substance removed. To this I declined, as there was already too great a disfiguration of the nose, without the addition of a cicatrix.

I then applied to another surgeon, a professor in a medical institution, and was advised the same operation, to be performed in the same way. I lastly repaired to Philadelphia, and consulted Dr. George McClellan, of the Jefferson Medical College. This gentleman, for the first time, pronounced it a congenital malformation of the septum narium. Upon examination, it was ascertained to commence where the cartilaginous septum joins to the vomer; thence inclining to the right of the mesial line, it completely obstructed the right nasal passage, giving to the external appearance of the nose a very marked lateral curvature. Professor McClellan commenced the operation of extirpating the septum by passing a sharp-pointed bistoury through its inferior portion, with the edge quite to the floor of the nostril; then adroitly curving and carrying it posteriorly nearly to the vomer, it was turned upwards and then forwards to the place of beginning, entirely removing the septum, without in the least interfering with either alæ.

Since that time, thanks to the skilful hand of the operator, my nose has become straight, my enunciation good, and I am enabled to breathe without an half-opened mouth. AN M. D.

November 1837. *Ibid.*

---

10. *American Crania.*—[At a late meeting of the medical section of the British Association, in Liverpool, Dr. Warren, of this city, made the following remarks, as published from the notes of a stenographer; but they are not, we are informed, exactly reported. Probably a more precise account will be published hereafter.]

Dr. Warren, of Boston, U. S., was called on for his paper on "Some remarks on the crania of the Mound Indians of the interior of North America, as compared with the crania of the South American Indians of Peru." As a stranger, though not exactly a foreigner, he felt it his duty, for the very valuable information he had received at the meetings of that section of the association, to make some contribution towards the facts which the British Association had so sedulously collected. There were some facts peculiar to that part of the world in which he resided, which, of course, could not come within the cognizance of the members of the association, and he would endeavor to state them; whether they would be considered important or not would be for their consideration. A considerable number of years ago, he accidentally came into possession of a cranium which struck him as an extraordinary one, and on examination he found that it differed from the crania of the well-known races, and the individual nations composing those races. He was led by this to make some inquiry into its history, and he ascertained that it came from the banks of the Ohio river, far back in America, in what was called the Western Country, and that it was discovered in a cavern on the top of a high and almost inaccessible rock, at the distance of about forty or fifty feet from the banks of the Ohio, by some hunters pursuing an animal, which took refuge in the cave. They there found the scull and the other bones of the body, in a fine state of preservation. The bones were so situated that they might have been there for several ages without decomposition, the calcerous rock which formed the bottom of the cavern absorbing all moisture. From the inaccessibility of the place in which they were found, it was probable that they had remained there for centuries. It was natural to suppose that this head must have been one of the aboriginal Indians of North America; but, on examination, he ascertained that such was not the fact, its whole structure being different from the Indian crania. He suspected that it might have connection with those races which had been discovered to be deposited in the ancient works or mounds of North America, and he soon obtained heads from that part of the country, and satisfied himself of the fact. He would presently state the particulars in which they differed from other heads. They had frequently heard of the mounds in the interior of North America. They were exceedingly curious, and many of them were found in wilds which had scarcely been trod by the foot of civilized man, and were covered with immense forests. They found elevations of earth which were quite extraordinary, and would be so even in any country. These mounds were covered by immense trees, and the observer was struck at once with the great antiquity which must belong to them. There were different kinds of mounds. There were some which had a great resemblance to fortifications, regularly made and of considerable extent. There was one at Circleville which was more than a mile in circumference, and which was surrounded by a wall or fortification, about thirty feet in height, with regular openings in different parts of it, and these openings guarded by interior works, similar to those in fortifications in the present day. These mounds were generally situated near the confluence of important rivers; there was one at the conflux of the Ohio and Muskingum rivers; they were so situated as to command the passage of the rivers. The mounds were perfectly regular, flat on the summit, and frequently a sort of excavation on the top of the summit. They were partly intended for the purposes of interment, and partly for places of worship; and probably the excavation found on the summit was a place where human sacrifices were made. Some of these works were very similar to the great temple at Cholula, in South America. These works were of very great extent, extending a length of 1000 miles, from the banks of the great lakes in Canada to the Gulf of Mexico, and filled all the most fruitful parts of North America. The heads he had spoken of as having obtained were taken from one of these circular mounds. The head in question, in common with all heads taken from these mounds, differed from the Indian and European formation. There was less extension of forehead than in the European head, but it resembled it; the elevation of forehead being equal to the *Caucasian* race. The vertex also was uncommonly elevated. The seat of the organ of veneration seemed to be very much developed, and it was evident that they were a very religious nation, for there was evidence that they made many human sacrifices. The formation of the skull approached to the Peruvian. But the most remarkable fact was the flattening of the *occiput*, which gave the *cranium* a peculiarly rounded form, and some even were quite circular. The occiput also was almost always more flattened on the right side than on the left. Another peculiarity in these heads was that the palatine fossa was of a rounded form. The lower we descended in the scale of races, the nearer we approached the animal formation. They knew that in the animal formation the jaws were very elongated, which gave animals greater perfection in taste and smell. There was an approximation to the African race in a small degree in the North American Indian; but as we rose to the Caucasian race the palate was shorter and smaller; so that taste and smell were inferior in the Caucasian races. Animals probably had a power in discriminating noxious smells and herbs, which we had not.

After he had been in possession of these heads for a number of years, he was anxious to generalize his remarks. When he was expecting contributions from the interior part of the country—for the mounds were situated very far from the part where he lived—say a thousand miles—it was difficult also to obtain these bones, as many of them were found in a state of decomposition—he found, one morning, three heads lying on his table. He immediately examined them, and supposed them to be skulls of the Mound Indians. But a few days after, the gentlemen who had furnished them came to Boston, and said to him that these were the heads of Peruvians, and that they were taken from an island near the city of Lima, a place renowned amongst Peruvians, where Mango Copac was said to have descended from the Sun, in order to enlighten the Peruvian race. He afterwards showed the skulls to Dr. Spurzheim, and he said they were all precisely of the same race. He perceived that the organ of constructiveness was peculiarly developed in all these heads. Inquiring further into the history of the Peruvian heads, he found three descriptions; one similar to the one he had been describing, having a flattened occiput, temples wide, and forehead particularly elevated. But there was another description much more common, which was of an oblong form, and very much resembled an egg in shape. In this, the occiput, instead of being compressed and flattened, was very prominent indeed. Then, there was a third kind of Peruvian head, which did not exhibit any marks of compression. The first kind were all remarkably irregular, and wanting in symmetry. These heads had evidently been artificially flattened on the occipital and frontal part, and were well known to belong to the Inca race of Peruvians, as they were taken from the place where they were buried; and they also had some specimens of the people amongst them.

[The lecturer here described that a whole family of the noble race of Inca had been buried with their clothes, and every part of them in a surprising state of preservation, just as they lived before the Spanish conquest. The tomb in which they were found was circular, like a well, lined with bricks, and near the bottom a flat stone was put down, supported at the sides like a floor, leaving a large cavity underneath. The bodies were then put into the tomb upon this stone, and loose earth thrown over them. The cavity underneath the tomb drained off the water and damp, and thus the bodies were preserved.]

Having traced the exact similarity between the Mound skulls and the Peruvian skulls of the Inca race, the conclusion was irresistible, that these two people had a similar origin. Now they were situated at a distance of 1000 or 1500 miles from each other, and the heads of the intervening nations were entirely different from the one or the other. At first this appeared to him very extraordinary. And here he might remark on the great importance of investigations by anatomists to point out the history of those nations which tradition did not hand down. There was a race between these two races, and they had heads almost as flat as a pancake.

[A Peruvian head was here exhibited, which had been subjected to artificial compression, and which was nearly square, being perfectly flat behind, and nearly so on the forehead.]

He must say, for the benefit of phrenology, that so far from the intellects of these flat-headed persons being inferior, the Indians who possessed them were quite equal in intelligence to others of the same nation. He had the head of a celebrated chief, who had a most extraordinarily flattened forehead, and he was known to have remarkable talent. In fact, no person was thought of any consequence in that country, unless he possessed a flat head.—[A laugh.] They then legitimately inferred, that these two nations were closely allied to each other—that was, the nations who had inhabited the mounds, and the Peruvians, because there was no resemblance between the heads of these nations, and any other heads that were known.

He might conclude, with just intimating that there had been observed to be a resemblance between these two sets of heads, and the heads of the Hindoo race; the same rounded form, and similar smoothness in the bones of the head and face. The conclusion drawn was that the race of the Mound Indians was entirely dissimilar to the North American Indians; and second, that they were entirely similar to the Peruvian race, which would lead to the inference that these two were one race. The American Indians, he thought, had emanated from two different sources, one from the south part of America, and the other from the North West Coast.

---

11. *Nitrate of Silver in immense doses.*—There resides in the vicinity of London, a gentleman whose face must be familiar to many thousands of its inhabitants. His skin is intensely blue, from the internal use (or abuse) of nitrate of silver. He is now about 73 years of age, and at the age of 45 became affected with epileptic fits of the most violent and distressing kind. In these attacks he struggled so strongly that it required two or three people to hold him. The fits also were very frequent, and in one of them he totally and forever lost the sense of hearing. A most distressing noise in the head, however, has ever since harassed the patient. After trying various remedies for the epilepsy, without the least benefit, he was advised by the late Dr. Curry, of Guy's Hospital, to enter on a course of the nitrate of silver, beginning with small doses, a quarter of a grain three times a day, gradually increased. In the course of a few weeks the attacks began to diminish in intensity, and in a few months, the intervals became much lengthened. The medicine was continued in augmenting doses till the epileptic paroxysms entirely ceased, which they did within six months from the commencement of the course of medicine already mentioned. Dr. Curry never warned the patient as to the effect of nitrate of silver on the skin, if long continued, and the physician himself dying about this time, the patient persevered with the medicine for the space of three whole years, the dose being eighteen grains a day for nearly all the third year! The patient, who is a highly intelligent man, avers that he never experienced any unpleasant effect from the medicine—indeed, he felt no effect at all, except its remedial power in arresting the epilepsy. Not having any idea of its physiological agency on the skin, he continued the nitrate with the view of effectually guarding against the return of the malady which he so much dreaded. It does not appear that the blue color made any serious advance till towards the close of the third year, otherwise it is probable that he would have taken alarm, or that he would have been apprized of the circumstance by his friends. At this remote period, however, he cannot distinctly recollect the exact time when the blue tinge of the rete mucosum commenced or acquired its complete acmé of intensity. This fact is of considerable interest, as there is probably no case on record where such a quantity of the nitrate of silver has been taken, or continued so long. It accords with our own experience as to the absence of any sensible physiological effect on the stomach and bowels which usually attends the administration of this medicine—unless we except a certain soothing agency—probably the consequence of obtunded sensibility in the gastro-intestinal nerves and tissues. In all cases excepting epilepsy, however, it is never necessary to give more than a grain or two of the nitrate daily (pills made up with extract of liquorice) and not continued beyond a few weeks at one time. Combined with the compound extract of colocynth, also, it forms an excellent remedy for irritability of the primæ viæ attended, as is often the case, with torpor of the bowels and vitiated secretions.—*Brit. and For. Med. Rev.*

---

12. *Sir F. Smith on Creosote as an external application.*—The observations of Sir Francis Smith were originally read to the College of Physicians in Ireland, and have been published in our esteemed contemporary of Dublin, May 1837.

Sir Francis observes that practitioners in this country evince an indisposition for the use of new remedies—an indisposition, which, he thinks, amounts to a vice, although in its origin a virtue. We feel some doubts of the correctness of Sir Francis's opinion on this point. We see, in practice, in London, little evidence of the bashfulness which he deplores, nor of that coy reluctance to prescribe new drugs, which in Ireland would seem to amount to a vice; nay, could we trust the evidence of our senses, we should rather deplore the wholesale manner in which the new medicines are abused, and the want of discrimination, we might sometimes say of common sense, which is evinced in their selection or their distribution.

Sir Francis has not made many experiments on creosote, and those experiments have been confined to its external application. He observes that one source of discrepancy in results will exist in the difference of specimens made use of; the strength or concentration differing much both from the variety in the mode of preparation and purification, and also from the mode of preservation. That which he has always used from the Apothecaries' Hall, of sp. gr. of 1,064, and nearly colorless. He has tried the creosote in three cases, or rather, he relates three cases in which he has tried it.

*Case* 1. A gentleman had phymosis, with the outside of the prepuce covered with ulcers of a ragged phagedæmic appearance, with a good deal of constitutional disturbance. Some alterative doses of blue pill in combination with morphia and tartar emetic, were prescribed, with soothing applications to the parts. This treatment had the effect, in conjunction with the recumbent posture, of quieting the constitution, and diminishing the tension of the parts. But every means was tried in vain to cause the ulcers to heal; they still preserved their phagedænic character, and at length united into a zone, nearly surrounding the prepuce, and threatening its destruction. Sir F. Smith now applied the creosote pure with a pencil. Next day the sores were improved, and had contracted one-third in diameter. Being lightly touched every day, for six days, they were entirely healed.

*Case* 2. A young gentleman, much broken down in constitution, became affected with an abscess by the side of the anus, which opened near the latter. On passing a probe into the orifice, says our author, "I found an orifice through which the matter, which amounted to an egg-cup full, had been evacuated; and in passing in a probe, I found it to enter about one inch, passing upwards, and resting on the side of the gut; on further investigation, I found that the sinus branched about midway its course into another cul de sac, which passed rather away from the gut, and presented to the probe a rough, yielding, spongy sensation; at the opposite verge of the anus an inflamed pile existed." The soothing plan was continued, but did not answer; under these circumstances, the creosote was resorted to. Sir F. introduced to the bottom of each cul de sac a very small dossil of lint smeared with it. In two days more the creosote was repeated, and four more applications spread over a fortnight, nearly perfected the filling up the sinus. The red precipitate afterwards completed the cure.

*Case* 3. A lady of a scrofulous habit, had had for some months ulcers of the septum narium, which had resisted much and varied treatment. Sir F. resolved to do nothing but employ the creosote locally.

"The ulcers amounted to four, varying in size from the head of a pin to the section of a very large pea; three on one side of the septum, and one (the largest and the first which had made its appearance,) on the other side.

I at first made use of a wash, consisting of one part creosote, with sixty of water; in which proportions they unite at common temperatures; the wash to be snuffed up the nose frequently in the day. The odour was complained of as very disagreeable, and at the end of two days, I was disappointed by finding that I had made no progress in improving the appearance of the ulcers. I now determined to apply the creosote in its pure form, and began by pencilling the edges of the ulcers with a brush smeared with creosote, and directing the patient to inhale the fumes of acetic acid for a few seconds subsequently. The application of the pencil was rendered easy by firmly grasping the ala nasi, and drawing it outwards; and I advised the inhalation of the fumes of acetic acid for two reasons; first, because acetic acid is the proper solvent of creosote, and would, by being inhaled immediately after its application, have the effect of rendering its action more equable and uniform; and secondly, because the odour would tend to counteract the disagreeable fuliginous flavor of the creosote. The next day I had the gratification to find the character of the ulcers improved; the edges were much less abrupt, and I now determined to apply the creosote lightly over the whole of the ulcer on the left side of the septum, and to brush those on the right side with a solution of creosote in twenty parts of acetic acid; and I continued to do so on alternate days for a week, at the end of which time, the ulcer on the left side of the septum was reduced to a mere point, having every appearance of immediately healing; whilst those on the right side, though improved in appearance, having smooth edges gently declining towards the centre, still preserved their original dimensions. I now applied to them also the pure creosote, repeating the application on alternate days, accompanied with the inhalation of the fumes of acetic acid. The rapidity with which the ulcers now healed was truly wonderful. That on the left side, to which I first applied the creosote in a pure state, was completely healed in ten days, and those on the right side, in six days after the application of pure creosote, and sixteen from commencement of treatment."

Sir Francis observes, that for small ulceration in the mucous membrane the pure creosote is most effective—that the solution of one part in twenty of acetic acid is not destitute of power—but that the solution of one part in sixty of water is only adapted for ulcers where the surface is large.

There can, we think, be little question that the creosote is an useful application where a powerful stimulant is wanted. It must be classed as a local agent with the balsams, the compound tincture of benzoin, &c. Probably those surgeons who possess the greatest amount of experience will be least disposed to attach to it any very marvellous properties.—*Med. Chir. Rev.*

---

13. *On the treatment of Ileus with Belladonna Clysters.* By Dr. Wagner, District Physician at Schlieben.—The following cases are given in illustration of the treatment recommended by Hanius.

*Case* 1. On the 21st of April, Dr. Lohrenz, of Schonewalde was called to visit a man, aged twenty-three, who had been complaining, since the 19th, of violent pains in the umbilical region. The pains came on periodically, and were greatly exacerbated by pressure, so that the patient screamed out when touched. He had incessant retching, his belly was hard and tense, and he had been several days without an alvine evacuation. Venesection, leeches, enemata, and various other external and internal remedies, were employed without any effect; his symptoms increased in intensity, and on the 22d, he had subsultus, syncope and vomiting of feculent matter. His belly was tympanitic, hard and painful, his bowels obstinately costive, his pulse scarcely to be felt, his anxiety intolerable, and his body covered with a clammy sweat. Under these circumstances, Dr. Lohrenz had recourse to clysters of belladonna. One half of the lavement was first injected; and unlike the other enemata, which were almost immediately rejected, this was retained, and had a marked effect in calming the violence of the patient's symptoms. His countenance became more cheerful, and his abdomen softer, but the pupils became greatly dilated. Half an hour afterwards, the second half was injected, and produced the most decided improvement. It was speedily followed by copious evacuations from the bowels, the pulse rose, the pain and vomiting ceased, and next morning the patient felt quite restored, and has not had since that time any return of his complaint.

*Case* 2. On the 4th of June, Dr. Wagner was called to see a laborer's wife, aged 40, of spare habit, but otherwise robust and healthy. The patient complained of a violent cutting sensation in the bowels, with obstinate costiveness and incessant vomiting. She had had repeated attacks of the same description before, but much milder, and of brief duration. On examination, he found a hernial tumour in the right groin, about the size of a walnut, and so excessively tender on pressure, that she could not bear the slightest touch. The belly was tympanitic and tender, the pulse small and rapid, the face pale, the body moderately warm. A large venesection was premised, and all the usual internal remedies (except quicksilver) tried without any effect; clysters of all kinds were employed, but proved equally ineffectual. The patient refused to submit to a second venesection or the application of leeches, and rejected altogether the proposal of an operation. On the 5th, all her symptoms were increased; her thirst was excessive, and she had fecal vomiting, with suppression of urine. In this state of things, Dr. Wagner had recourse to the belladonna clysters. He infused a drachm of the root of belladonna, and an ounce of chamomile flowers (he does not state how long) in twelve ounces of water, and divide the infusion into three parts. The first part was administered by himself as soon as it was cold, and produced very remarkable effects. The nausea and vomiting instantly ceased, and half an hour afterwards, the belly was soft, without much tenderness on pressure, and the hernial tumour much less tense, though still painful. None of the secondary bad effects of belladonna were observed. On visiting the patient at noon, he found her quite easy and contented, but laboring under dilatation of the pupils. She told him that she had been threatened with a repetition of the attack about half an hour before, but she had stopped it *by drinking a few spoonsful of the clyster mixture.* In the evening, when seen by Dr. Wagner, she complained of a return of the abdominal pain and tension, and as there was no indication of the secondary effects of the belladonna, except some dilatation of the pupil, he administered the remainder of the infusion. The patient passed a quiet night, with the exception of some troublesome dreams, and, on the following morning, the abdominal symptoms were mild and inconsiderable, except that the hernial sac remained extremely tender on pressure, and the incarcerated portion of intestine could not be replaced. At noon, the soreness and tension of the belly increased again, and as no alvine evacuation had as yet taken place, and there were no apparent bad consequences from the belladonna, Dr. Wagner repeated the infusion as before. The first dose produced the usual tranquilizing effect; but no further change; and as the constitutional effects of the remedy were limited to some increase in the dilatation of the pupils, with unpleasant dreams, he administered the second portion, and, towards evening, the third and last.

On the morning of the 7th, the hernial tumour had disappeared, loud borborygma were heard in the abdomen, and large evacuations of offensive fæces took place; but the patient, after having been annoyed the whole night with frightful dreams, was suddenly seized with such furious delirium, that it required several strong men to hold her. Her eyes was fixed and sparkling, the pupils excessively dilated, the conjunctiva injected, the cheeks of a fiery red, the pulse small, rapid, and scarcely to be felt, *deglutition unimpeded.* She saw nothing but strange phantoms, which she sought to drive away by abuse and threats, and searched for concealed enemies under her bedding, clothes and furniture. She believed herself perfectly well, wished to resume her domestic labors, pulled on her clothes with furious violence, and would have rushed out of the house had she not been held by force. Dr. Wagner ordered enemata of vinegar, (which were followed by copious evacuations,) and gave vinegar with strong coffee internally, of which the patient drank large quantities with much desire. Cold lotions were applied to the head, and the limbs were washed with vinegar, an operation which the patient herself performed with apparent satisfaction, washing herself with vinegar from head to foot. This state of things continued until the morning of the 8th, when the patient became rational and composed, but complained of flashes of light and various other optical phantasms, with a sense of great weight and pressure in the head, and a general feeling of soreness and exhaustion, particularly in the feet. She recollected distinctly every thing she had said and done during the preceding day and night, and said that the horrible phantoms by which she was incessantly surrounded had compelled her to act and speak in the manner she had done. On the ninth, she complained of nothing but weakness; which soon disappeared, and she recovered rapidly, without any farther unpleasant symptoms.

*Case* 3. On July 3d, a smith, aged 59, was attacked with enterodynia, vomiting, tympanitic swelling of the abdomen, and constipation. Dr. Wagner was called to see the patient, and found an incarcerated hernia of the left groin, about the size of a hen's egg, and extremely sore to the touch. All external and internal remedies, repeated local and general bleeding, and frictions over the abdomen with extract. belladonnæ and ol. hyoscyami, proved wholly ineffectual. Every thing was instantly vomited up, and the clysters were immediately returned. As the patient would not submit to an operation, Dr. Wagner threw up an enema, composed of a scruple of the belladonna herb, and half an ounce of chamomile flowers, in four ounces of water, which arrested the vomiting immediately, and produced such a diminution of the pain, that the patient was able to enjoy several hours' sleep. The abdominal symptoms, however, returned every six or eight hours, and were four times allayed by the use of the same enema. On the 5th, return of the pain and tenderness; Dr. Wagner was afraid to have recourse to the belladonna, as, in addition to great dilatation of the pupils, frightful dreams, sinking and acceleration of the pulse, and dryness of the tongue, had taken place; and he prevailed on the patient, after much entreaty, to submit to the operation. This was performed successfully by Dr. Weistand, on the 6th, and in fourteen days the patient was quite well.

*Case* 4. On the 5th of July, Dr. Wagner was called to a woman, aged 47, who was said to have been laboring for two days under violent pains in the abdomen, obstinate constipation, and incessant vomiting. On making an examination, he found an incarcerated femoral hernia of the right side, about the size of a small walnut, and excessively tender to the touch; diffused abdominal tenderness and tympanitic distention. Bleeding, leeching, frictions over the abdomen with belladonna and hyosciamus, and various other remedies, were employed without any effect; and the symptoms assumed a very alarming character. As the patient refused to submit to an operation, Dr. Wagner had recourse to the belladonna clysters, which produced the usual tranquilizing effects, but the hernia remained irreducible, and the patient began to exhibit some of the symptoms of poisoning, as dilatation of the pupils, sparkling of the eyes; a fiery red color of the cheeks, and acceleration of pulse. Blood was now drawn from the arm, small doses of calomel and laxative salts given internally, and the belladonna clysters continued, until six lavements (each composed of ℈i. of belladonna to ℥iv. of water) were used. The hernia, however, remained irreducible, and as the patient would not submit to an operation, Dr. Wagner discontinued his visits on the 8th. On the 9th, however, the greater part of the hernial tumour had disappeared, the patient had several copious stools, and in the course of two days, found himself quite well.

[The foregoing cases show that belladonna, like tobacco, is a remedy of great efficacy in subduing symptoms of ileus connected with incarcerated hernia. It has also the advantage of relieving pain, without substituting for it the horrible sickness and sinking of the vital powers, which results from the use of tobacco. Two facts, however, connected with the history of belladonna, will always tend to diminish its applicability; namely, its tendency to accumulate in the system, and then explode with fearful violence, and the well known fact, that its specific powers vary in a remarkable degree according to the place in which it grows.]—*Journal de Pr. Heitkunde. August*, 1836.—*Brit. and For. Med. Rev.*

—

14. *On St. Vitus's Dance.* By Dr. Stiebel.—Nearly one hundred cases of this disease have come under the notice of Dr. Stiebel; and in not one of these, he says, was wanting the evidence of an irritation of the spinal nerves. Few of the patients, during the course of the disease, have not had pain in some one of the vertebræ, and all, either in consequence of the treatment employed or spontaneously, have recovered. Dr. Steibel believes that the cause of this disease is an irritation of the motor nerves of the spinal marrow or of the medulla oblongata, depending on inflammation or turgescence. Chorea almost always originates during the development of the spine and of the spinal marrow; generally between the seventh and seventeenth years, but occasionally at later periods of life.

The following appears to be the anatomical explanation of the disease:—The spinal marrow, and the origins of its nerves, lie within a bony cavity. If, during their development, there occurs a want of relation between the bones and the nervous system, so that the cavity does not correspond to the increasing marrow, the nervous origins become subject to an irritation as of a foreign body. This disproportion may be the effect of swelling of the spine, without previous change in the nerves, as well as of turgescence of the membranes of the nerves and the nerves themselves, the spine remaining unaltered; but the first is of most frequent occurrence. The spasms generally cease during sleep, although the irritation continues. The chorea may be limited to one side of the body, or to parts of less extent, according to the situation of the irritation. It is liable also to change its place, to become general after having been but partial, and *vice versa*. It may also alternate with paralysis, which is as little dangerous as the original disease. The change of locality of the disease is not sudden, as in hysteric spasms, but at different periods of the progress of development, each of which is of a certain duration.

Partial chorea of individual motor nerves of the organs of sense constitutes some of the most remarkable forms of the disease; such, for instance, as a constant twitching of the eyelids, distortion of the eyes, abnormal motions of the tongue, and almost uninterrupted sneezing.—A very common form of partial chorea consists in the constant and unavoidable utterance of inarticulate sounds. As all the motor nerves of the organ of voice are not affected, the patient can still speak, but with effort and stammering, the inarticulate sounds being afterwards made more uninterruptedly and with greater violence. This utterance of sounds frequently continues for weeks, only interrupted during sleep; a long-continued aphonia frequently follows, or a shooting pain on one or the other side of the breast, or asthma. Palpitation of the heart accompanies chorea, both partial and general. In examining individuals suffering from either local or general chorea, it will be very rare not to find tenderness and swelling of some vertebral bone; but such an examination must be repeated, as the sensibility is frequently not discoverable until the disease has lasted for some time. In all the cases of chorea which Dr. Stiebel has examined, he has discovered no other cause than that above mentioned, although he does not deny the possibility of the existence of other causes;—e. g. metastasis of rheumatic inflammation, injuries of the spine, &c. There is doubtless an hereditary disposition to chorea, it having been known to affect whole families. It is particularly frequent among the Jews. Dr. S. has not himself had any opportunity of examining the bodies of individuals who have died of chorea; but, in all the cases which he has read, changes in the spinal marrow, its membranes, or in the spine, are mentioned.

The treatment of chorea is simple. Leeches, followed by mercurial inunctions, and then by more active exutories, are indicated when a painful vertebra is found to exist. If no tender point is at first discoverable, leeches and blisters may be applied along the spine. Calomel is generally given internally as a derivative. If the disease is not thus relieved, repeated cold douche-baths on the spine are almost always of advantage. As a general rule, the disease is cured between the fourteenth and twenty-first day by this treatment; but, should not this happen, the disease may be left to nature, which almost always put an end to it in the progress of development. At the same time, however, it is necessary to guard against the occurrence of any spinal deviations, and irritant frictions may be applied to the spine. Preparations of iron have sometimes appeared to be of use. The description of the disease which is here given will suffice to account for the credit which such numerous remedies have acquired in the cure of chorea; their use having been coincident with the natural cure. It is not intended to deny that there may be a specific remedy, but only that we at present know not what it is.—*Wochenschrift fur die gesammte Heilkunde.* No. I. 1837.

—

15. *On the first changes in the Ova of the Mammifera, in consequence of impregnation; and of the mode of Origin of the Chorion.* By Thomas Wharton Jones, Esq. *Read before the Royal Society, April* 27, 1837.—The author having, in a former paper, described the structure of the unimpregnated ovum of mammiferous animals, now proceeds to investigate the changes which the ovum undergoes in consequence of impregnation. In the rabbit, the first perceptible difference is the addition of a thick gelatinous matter surrounding the parts of which the ovum was composed in its original state, and apparently derived from the ovaries. In the progress of development the vitellary membrane gives way, as happens in the ova of the newt, and of many of the oviparous animals. The gelatinous envelope acquired in the ovary, and which is more especially circumscribed and defined after impregnation, constitutes the only covering of the vascular blastoderma, after the giving way of the vitellary membrane, and afterwards forms the chorion, which in rodent animals, at a further stage of development, presents itself under the form of a thin and transparent membrane, very similar to the vitellary membrane of a bird's egg, and situated immediately outside the nonvascular and reflected layer of the umbilical or erythoid vesicle. The author draws similar conclusious with regard to the development of the human ovum.

The second part of the paper relates to the changes taking place in the vitellus, the inferences concerning which are deduced chiefly from observations of the development of the ova of batrachian reptiles. The author concludes that the disappearance of the germinal vesicle is prior to impregnation. In the newt, the vesicle, at first imbedded in the substance of the yelk, gradually approaches the surface, until its situation is immediately underneath the vitellary membrane: its coat, having now become very soft, gives way, allowing the contained fluid to be effused on the surrounding surface of the yelk; and the small depression in which the vesicle was lodged now forms the cicatricula. The effused fluid gives a degree of consistence to the matter composing the surface of the yelk, and thus promotes the formation of the blastoderma. In the frog, the surface of the yelk becomes every day more and more broken up, and the resulting crystalline forms described by Prevost and Dumas, become smaller and smaller, until the surface of the black blastoderma appears under a magnifying glass like shagreen. The blastoderma, consisting of an aggregation of clear globules, different from those of the rest of the yelk, is now fully formed, and has extended itself so as to close in the white spot. The change which takes place in the yelk of the bird's egg appears to be limited to the neighborhood of the cicatricula.—*Proceedings of the Royal Society*, No. 29.

